# The spinning apparatus of webspinners – functional-morphology, morphometrics and spinning behaviour

**DOI:** 10.1038/srep09986

**Published:** 2015-05-07

**Authors:** Sebastian Büsse, Thomas Hörnschemeyer, Kyle Hohu, David McMillan, Janice S. Edgerly

**Affiliations:** 1 University Museum of Zoology, Department of Zoology, University of Cambridge, Cambridge, UK; 2 Johann-Friedrich-Blumenbach-Institute of Zoology and Anthropology, Department of Morphology, Systematic and Evolutionary Biology, Georg-August-University Göttingen, Göttingen, Germany; 3 Department of Biology, Santa Clara University, Santa Clara, CA, USA

## Abstract

Webspinners (Insecta: Embioptera) have a distinctly unique behaviour with related morphological characteristics. Producing silk with the basitarsomeres of their forelegs plays a crucial role in the lives of these insects – providing shelter and protection. The correlation between body size, morphology and morphometrics of the spinning apparatus and the spinning behaviour of Embioptera was investigated for seven species using state-of-the-art methodology for behavioural as well as for morphological approaches. Independent contrast analysis revealed correlations between morphometric characters and body size. Larger webspinners in this study have glands with greater reservoir volume, but in proportionally smaller tarsi relative to body size than in the smaller species. Furthermore, we present a detailed description and review of the spinning apparatus in Embioptera in comparison to other arthropods and substantiate the possible homology of the embiopteran silk glands to class III dermal silk glands of insects.

In spiders (Arachnida: Araneae) silk production is well known and well studied. In this group silk is vital in nearly all biological contexts. For example, it is used for protection^1^, in reproduction and prey capturing^2^ as well as in dispersal^3^. However, all those applications of silk are known for insects as well, for example: protection in Lepidoptera and Embioptera^4^,^5^,^6^, reproduction in Neuroptera, Diptera and Zoraptera^4^,^7^,^8^, foraging in Trichoptera and dispersal in Lepidoptera^4^. Silk spinning in insects is common but remains comparably unnoticed and therefore barely studied^9^. A complex spinning behaviour as well as a social behaviour related to silk spinning as in spiders^10^ is, among adult insects, only present in the Embioptera^11^. Furthermore, a comprehensive description of the internal and external morphology is rather rare for insects compared to spiders, especially in combination with behavioural and ecological background.

Embioptera (webspinners) is a small group of ca. 360 known species^12^ belonging to the polyneopteren insects. Taxa that have been discussed as potential sister-groups include the Plecoptera^13^, Orthoptera and Phasmatodea (summary in^14^). Currently a sister-group relationship with Phasmatodea seems to receive the best support from molecular as well as from morphological data^15^,^16^,^17^,^18^,^19^,^20^,^21^,^22^,^23^,^24^.

Embioptera are most diverse in the tropical and subtropical regions but they also occur in the Mediterranean and in other semi-arid regions^6^,^12^,^25^,^26^. Recently Miller et al.^27^ presented a comprehensive analysis of the phylogeny of Embioptera using morphological as well as molecular data. Although a few of the historically recognized families were not recovered, several others gained strong support, including Clothodidae and Oligotomidae, the subjects of this paper. Even though the relationships between some families are not well resolved, the monophyly of the order is undisputed. Their distinctive tarsal silk glands are their best-known characteristics.

The spinning apparatus of Embioptera is composed of a more or less large number of individual glands (e.g. ca. 53 in male or 31 in female *Aposthonia ceylonica*^28^) that are arranged next to each other in each spatial direction within the basitarsomere of each foreleg. Each gland consists of a reservoir surrounded by gland tissue secreting silk into the reservoir. A secretory duct leads the secretion to the spinning field on the ventral surfaces of the basitarsomeres where it is ejected through hollow setae (silk-ejectors)^6^,^28^,^29^,^30^,^31^,^32^,^33^,^34^,^35^.

Embiopteran lifestyles related to habitat can be roughly divided into distinct and intermediate types. Some webspinners build their silken galleries under or on the bark of trees (Fig. 1). Others spin silken domiciles beneath rocks and logs and/or in leaf litter where the silk is often thin and sometimes coated with gathered materials as in *Haploembia tarsalis* (Oligotomidae) (Fig. 2). In addition, some species are opportunistic being found in either situation depending on the availability of water. When the environment is humid, their colonies are on tree bark; when dry, they are in leaf litter or in underground burrows. One opportunistic species, readily colonizing different microhabitats, is *Oligotoma nigra* (Oligotomidae). It was introduced to diverse habitats around the world via human trade and transport^36^. See Ross^6^ for a general review of lifestyles of webspinners and below for specific qualities of the species in this study.

Gross morphology of female Embioptera is remarkably homogeneous (e.g. see Supplementary Fig. 1). Adult females, who are juvenile in form, are so similar to each other that even specialists cannot discriminate them to family-level. Indeed, phylogenetic studies rely for the most part on male secondary sexual characters and wings, as well as on molecular evidence^12^,^27^. Females are wingless, elongate, flexible, and built for life in tightly spun silk tubes where they can execute rapid and tight U-turns. Given that different species dwell in different habitats and that the silk is the chief barrier between the webspinner’s soft body and its environment, it could be that subtle differences have evolved, not in body form, but in how they spin, produce, and use the silk. It is also possible that silk glands have diversified in response to selective pressures of microhabitats, although a recent overview of insect silks revealed a distinct lack of correlation between the use of silks and the diversity of the glands and proteins^9^. The diversity of webspinner silk glands was not known at the time that review was written and the question remains as to whether they fit the pattern detected by Sutherland et al.^9^.

A study characterizing silks from six species in five families of Embioptera revealed a consistent secondary structure of the silk proteins suggesting commonality of the amino acid sequence motifs^37^. On the other hand, recent observations of eight webspinner species in four genera revealed diversity in silk spinning styles^28^ (see Fig. 3 in^28^). The relationship of spinning behaviour to silk gland differences, however, has not been explored. The present investigation seeks to explore how silk gland morphometrics and silk spinning behaviour relate to body size. The Body Size Hypothesis states that body size dictates how much silk is produced, because larger webspinners need to invest more in silk and spinning to cover their bodies, which otherwise are conspicuous to predators, such as ants and birds^5^. Smaller webspinners can more effectively hide within bark crevices and within leaf litter. The Body Size Hypothesis predicts that large webspinners (1) will have larger silk glands (for producing more silk), more silk glands (for secreting more silk at a time) and/or larger reservoirs (for storing more silk), (2) invest in longer bouts of spinning, (3) produce more silk during the same amount of time, (4) have more complex spinning steps, and (5) spin more to the side, back and over the back (steps that create silk covering over the dorsum and around the body) (see^28^ for detailed spinning kinematic diagrams).

We here present an interdisciplinary study of Embioptera focusing on the elucidation of the complex morphology of the spinning apparatus and its functional correlation in the context of the sophisticated spinning behaviour. We investigate the diversity of silk gland morphology and to disclose correlations between morphology and body size. To achieve this, we combined approaches in comparative morphology, morphometrics and behavioural analysis. This interdisciplinary approach is a key feature of our project.

## Results

### General morphology of the spinning apparatus

A single basitarsal gland consists of two major structures, the reservoir, which is a lumen for storing spinning secretions and the syncytial gland tissue that surrounds each reservoir (Fig. 4A). During the spinning process the secretion is channelled through the so-called canal-cage^32^ a complex structure at the end of the duct; a structure that extends into the reservoir and into the associated duct (Fig. 4B, C). This duct transports the spinning secretion to the silk-ejectors at the ventral side of the basitarsomere where the secretion is ejected. Each gland has a single duct and silk-ejector. Therefore, the number of reservoirs should be identical with the number of silk-ejectors. Embioptera use their silk to produce galleries in which they live. They touch the ground with the ventral part of the basitarsus and release silk via the silk-ejectors. During the spinning process they show complex spinning steps moving their legs in any direction, performing wide arcs, as well as stepping above their bodies (especially over the tergum) to construct the ceiling of the gallery^6^,^11^,^28^.

### Species investigated

#### Aposthonia borneensis (Oligotomidae)

*Ap. borneensis* is a species from Southeast Asia that has spread more widely into urban environments than most other Embioptera. The colonies live on the bark of trees where they feed on lichens. They produce tube-like galleries that run along bark crevices. In laboratory cultures they also produce patches of silk where they cluster together. The tubes that are produced in laboratory cultures are thick and run up the sides of the containers. The spatial arrangement within the basitarsomere is compact. There is no visible lumen between adjoining glands. The reservoirs are voluminous. The diameter of gland tissue that surrounds each reservoir varies considerably (Supplementary Fig. 2A and 3A).

#### Aposthonia ceylonica (Oligotomidae)

The Southeast Asian *Ap. ceylonica* also has a more urban affiliation. *Ap. ceylonica* were collected in India where they usually live on the bark of trees, feeding on lichens. The behaviour in the laboratory resembles that of *Ap. borneensis*. The spatial arrangement within the basitarsomere also is rather compact. The glands are located close to each other. Small to major lumina are present, including a pervading lumen from distal to proximal (Supplementary Fig. 2B). The gland tissue surrounding a reservoir is quite voluminous and unchanging (Supplementary Fig. 2B and 3B).

#### Haploembia solieri and H. tarsalis (Oligotomidae)

*Haploembia solieri* and *H. tarsalis* (Fig. 2A) are known from the Mediterranean. Both species were until recently assigned to the same species, but phylogenetic analysis showed that they were different species: *H. solieri* being sexual, *H. tarsalis* asexual^38^. They live in leaf litter and underground in crevices where they spin silk to create domiciles that protect them during rain. In particularly hot months they retreat below leaf litter. They extend their silk into leaf litter, into the bases of grasses and onto lichens on the undersides of logs on the ground. Both species have been introduced from the Mediterranean to California where our study specimens were collected. *H. tarsalis* is known only from asexual females, which live alone or in small aggregations with few offspring. The sexual females of *H. solieri* live in larger aggregations associated with numerous young and males. Individuals of *H. solieri* spin more silk than *H. tarsalis* females, as observed in the field and in laboratory cultures. The arrangement of glands within the basitarsomere is compact. There are no lumina between individual glands in *H. tarsalis* but a few small lumina in *H. solieri*. A small lumen pervades the basitarsomere meandering from distal to proximal in both species. The volume of the gland tissue surrounding the reservoirs is unchanging (Supplementary Fig. 2C-D and 3C-D).

#### Oligotoma nigra (Oligotomidae)

*Oligotoma nigra* is from Afro-Eurasia and was accidentally introduced to North America. It is an opportunistic species, living underground, in leaf litter or in bark crevices on trees, depending on the humidity. If dry, it retreats. The spatial proportions within the basitarsomere appear almost tidy and organized (Supplementary Fig. 2E). The glands have only a few borders in common and are mostly all embracing surrounded by lumina of variable sizes. There is also a small lumen that pervades the basitarsomere from proximal to distal. The volume of gland tissue surrounding the reservoirs is variable and the tissue looks perforated in some places (Supplementary Fig. 2E and 3E).

#### Eosembia auripecta (Oligotomidae)

**Figure 1:**
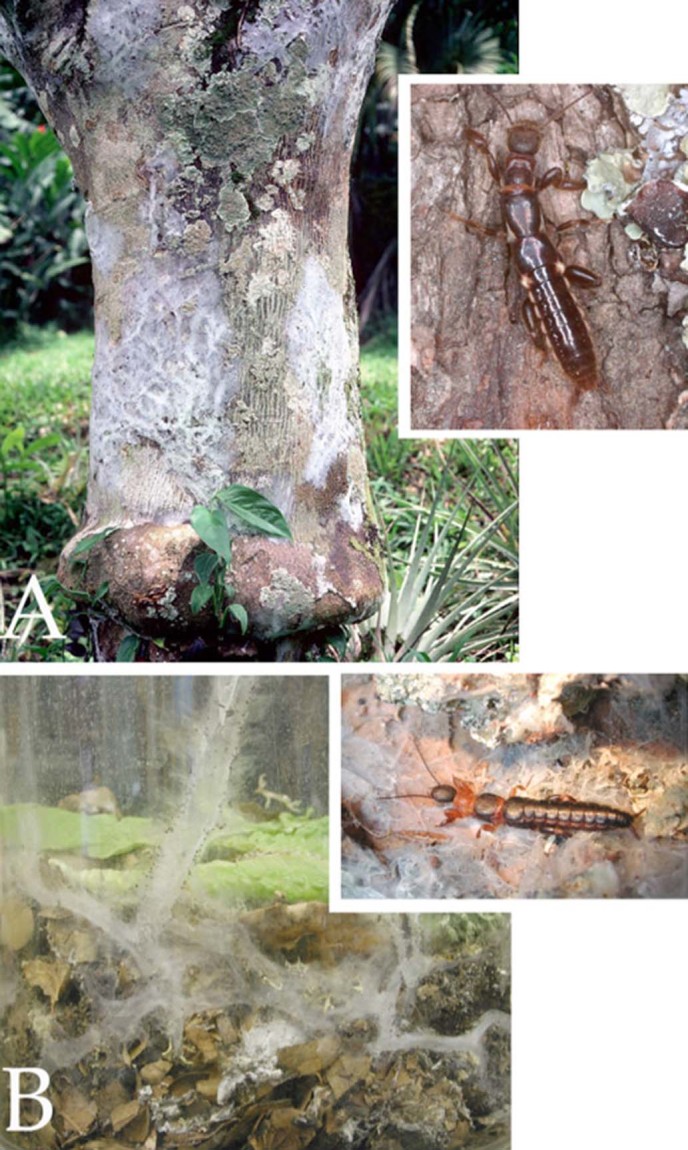
Embiopteran habitat. (A) Silk of *Antipaluria urichi* on bark of a citrus tree in Trinidad and an adult female (inset) approximately 1.7 cm in length. Silk covers the colony as well as the lichens upon which they graze. (B) Silk of a lab colony of *Eosembia auripecta.* The adult females (inset; approximately 1.8 cm long) live in leaf litter in the dry season and climb onto trees to breed during the more humid times of the year in northern Thailand. Silk tubes course through the leaves in this lab container and one tube extends up and out of the leaf litter, illustrating the products of their silk spininng. Photographs © J.S Edgerly.

**Figure 2:**
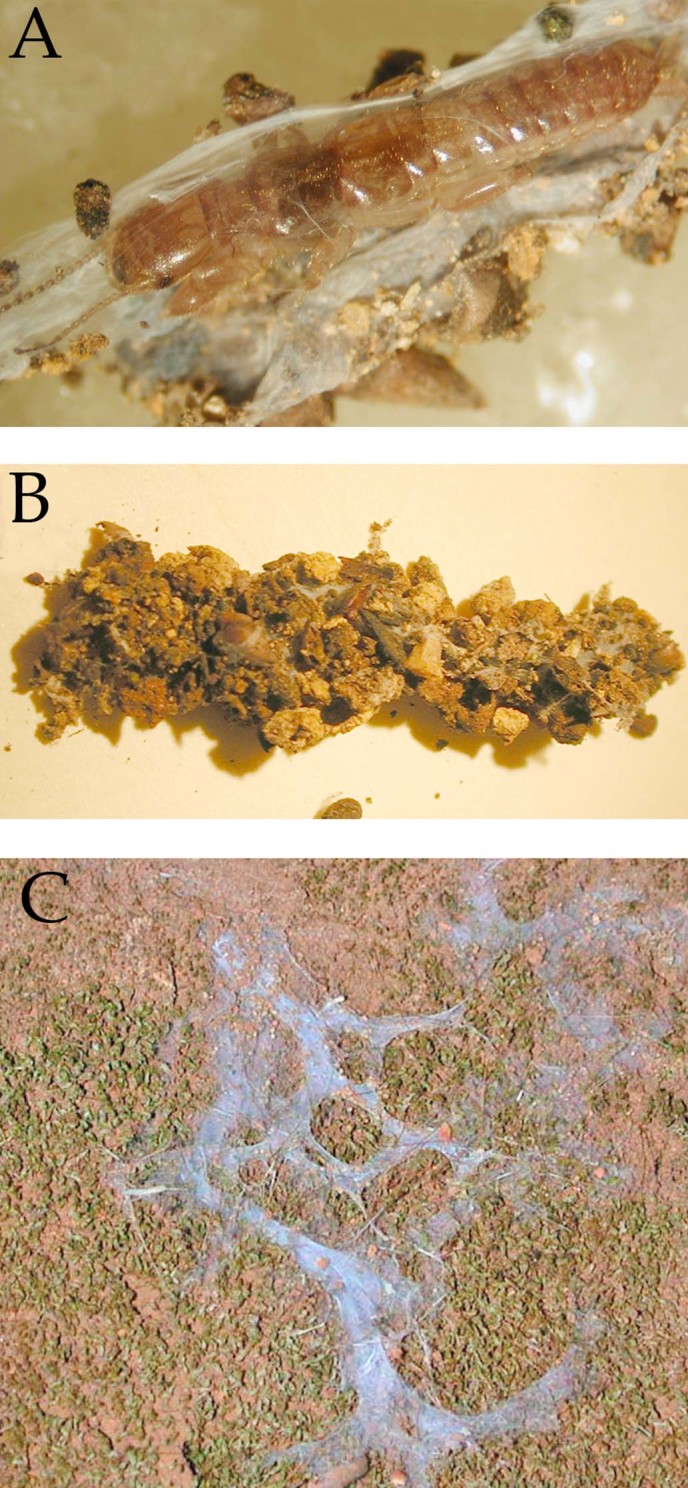
Embiopteran galleries (*Haploembia tarsalis)*. (A) An adult female (approximately 1.2 cm long) within a tightly spun silk tube, which was covered with gathered materials stuck to the silk (shown in B). (C) Silk in the field in San Jose, California. The individuals hide within burrows in the clay soil but they will also spin thin foraging tubes into the open or into leaf litter. Their silk is nautrally tinted blue. Photographs © J.S Edgerly.

*E. auripecta* (Fig. 1B) is a large embiid from Thailand. It is a productive spinner, and its colonies can be easily recognized on trees. The species is quite similar to *An. urichi* in terms of body size and silk production. Contrary to *An. urichi*, *E. auripecta* hides in the leaf litter as part of the life cycle. Hence, *E. auripecta* shows an interesting behavioural combination of hiding in leaves during the distinct dry season, and then moving up onto trees to reproduce and feed in the rainy season. The arrangement of glands within the basitarsomere is irregular (Supplementary Fig. 2F). The glands have no common borders but all-embracing tissue that is surrounded by narrow or wide lumina. There also is a small lumen that pervades the basitarsomere from proximal to distal. The volume of gland tissue that surrounds the reservoirs is quite constant and the tissue looks perforated (Supplementary Fig. 2F and 3F).

#### Antipaluria urichi (Clothodidae)

*An. urichi* is a comparatively large (Fig. 1A) and robust species occurring in neotropical rainforests in Trinidad where it lives on the bark of trees. It spins copious silk and constructs tubular galleries generally covered in several layers of silk (Fig. 1A). The spatial proportions within the basitarsomere are irregular, which becomes especially obvious in longitudinal section (Supplementary Fig. 2G). The glands are often surrounded by wide intercellular lumina. A large lumen pervades the basitarsomere from proximal to distal. The diameter of the gland tissue that surrounds a reservoir is more or less constant (Supplementary Fig. 2G and 3G).

### Test of the Body Size Hypothesis

Analysis of contrast scores of body length and size-corrected silk gland measurements (Fig. 5) revealed a significant positive relationship between body length and volume of silk reservoirs (*P* = 0.001). However, a negative correlation was found between body length and size-corrected tarsal length (*P* = 0.004). Size-corrected tarsal width was also negatively correlated with body length but was marginally not significant after a Bonferroni correction (*P* = 0.035). Larger webspinners have slightly longer tarsi in absolute terms but dis-proportionally narrower and shorter tarsi per unit body length when compared to the smaller webspinners (Fig. 5A and 5B). Independent contrasts of the behavioural parameters (spin time, step diversity and proportion side, back and over back) and area of silk produced were not significantly correlated with body length. Figure 6 displays the significant tarsal measurements, in absolute terms and corrected for body length, for the species arranged on the phylogenetic tree. The graph highlights the dramatic difference in the reservoir volume as a function of body length.

**Figure 3:**
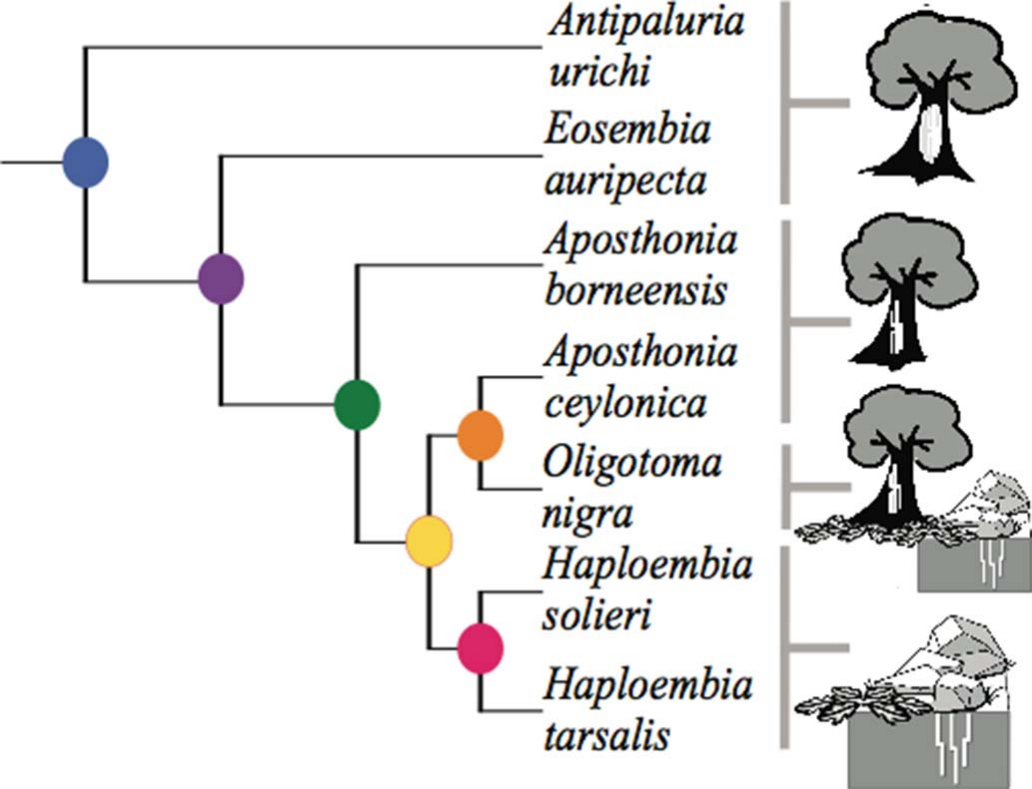
Simplified phylogenetic tree used in independent contrast analysis. See Methods for details of source of branching pattern. Icons represent basic lifestyles of the seven species, whereby (at top) the tree with a white patch shows more copious, sheet-like silk domicles, the tree (second from top) with distinct white lines represents arboreal species that line crevices in the bark with silk, the combination of tree, leaves and rocks (third down) reflects an opportuntistic lifestyle of *O. nigra*, and finally, the ground habitats of under rocks, within leaf litter, or underground represents the two more cryptic *Haploembia* species. Artwork © J.S Edgerly.

Two principal components axes captured most of the variation (77%) of the independent contrast scores [43.26% (PC1); 33.46% (PC2)] (Fig. 7, Table 2). Plotting nodes from the phylogenetic tree (Fig. 3) onto a PC graph (Fig. 7) shows a possible split in morphometrics and behaviour. PC1 describes proportion of spinning to the side, back and over the back, mean number of spin steps, and time spent spinning during the one-hour filming. PC2 describes area of silk spun in 24 h, tarsus length divided by body length, gland reservoir volume divided by body length, and body length.

## Discussion

The morphology and morphometrics of the spinning apparatus are elaborately described using synchrotron radiation micro computed tomography (SRμCT). The available µCT-data allowed for reconstructing the morphology of the gland system in the basitarsomeres in three dimensions down to the syncytial cell area, reservoir and ejection apparatus. Though the resolution of the µCT-data is at its limits here, it was possible to confirm an ejection apparatus with a “canal cage” that directly delivers the secretion via a duct, a possible end-apparatus, to a silk-ejector.

**Figure 4:**
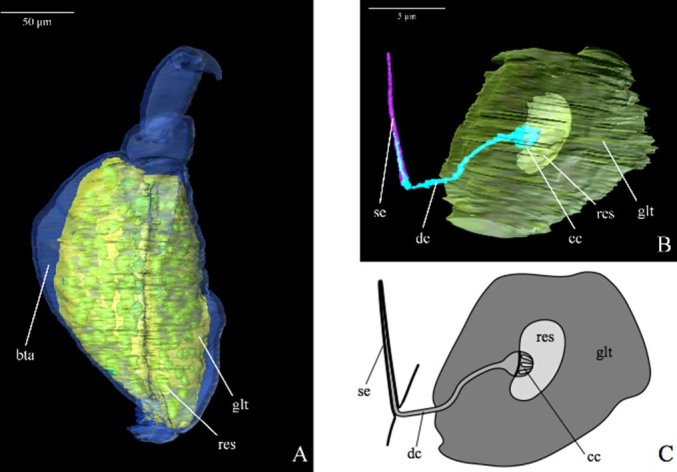
3D - reconstruction from SRμCT of *Haploembia solieri*. Bta - basitarsomer; cc - “canal cage”; dc - duct; glt - gland tissue; res - gland reservoir; se – silk ejector. (A) The spinning apparatus located within the prothoracic basitarsomer. (B) A single gland including the ejection apparatus. (C) Schematic drawing of a single gland.

**Figure 5:**
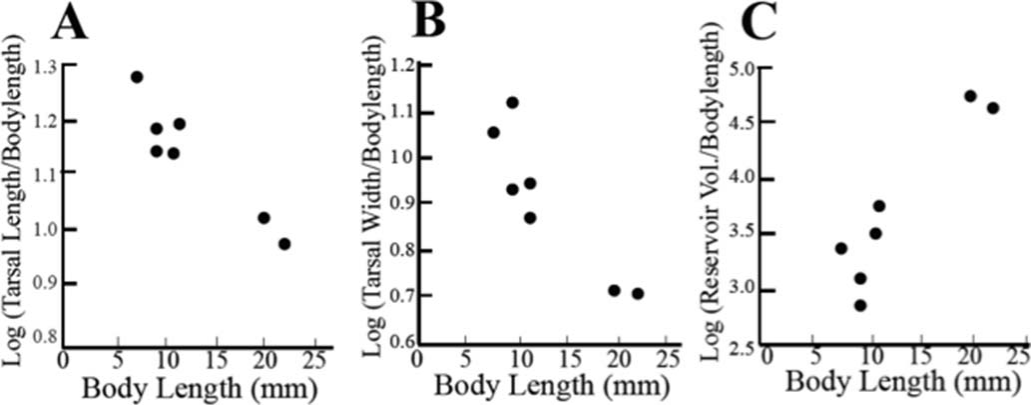
Scatterplots of morphological measurements of the basitarsomer and silk gland found to vary significantly with body length in a test of independent contrasts. (A) Log of tarsal length divided by body length, where “tarsal” is short-hand for the spinning tarsus (*P* = 0.004). (B) Log of tarsal width divided by body length (*P* = 0.035). (C) Log of reservoir volume divided by body (*P* = 0.001).

**Figure 6:**
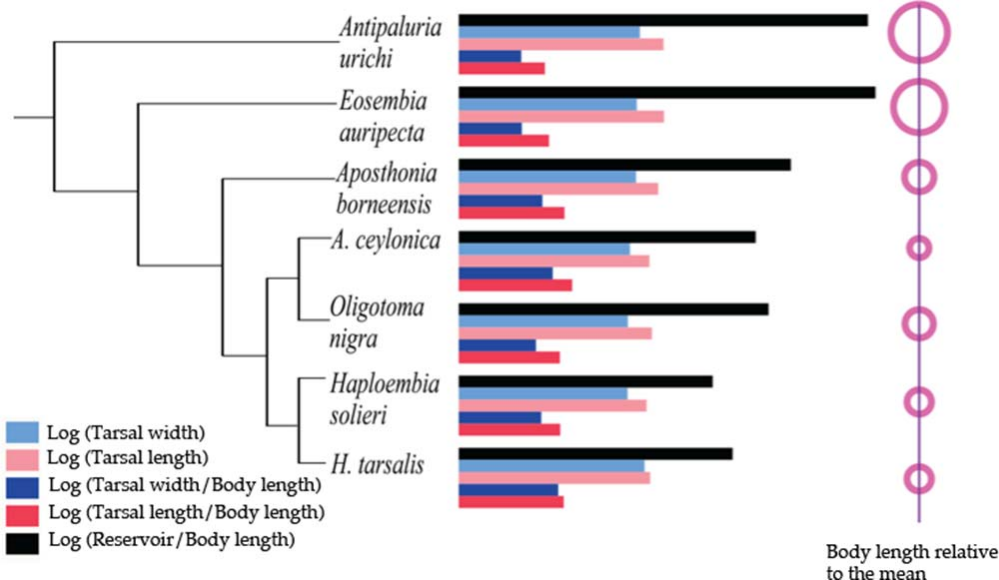
Phylogenetic tree with plot of means of the log of tarsal width and length, shown as absolute values and as corrected for body length. Reservoir volume divided by body length is also shown. Body length is depicted as relative to the mean size to highlight the relatively large size of *Antipaluria urichi* and *Eosembia auripecta*.

The first descriptions of the spinning apparatus of Embioptera by Melander^29^, Rimsky-Korsakov^30^ and Murkerji^31^ clearly stated reservoirs for storing the silk and “spinning hairs”, suggesting a connecting duct. Murkerji^31^ already mentioned the multinuclear character of the tissue surrounding a reservoir and Barth^32^ first mentioned the “canal cage” – a structure that intrudes from the duct into the lumen of the reservoir^32^ (Fig. 4B, C). Alberti & Storch^33^ and Nagashina et al.^34^ documented the multinucleated character of the silk gland surrounding a reservoir as well as the “canal cage” in great detail through studies of the ultrastructure. Dubitzky & Melzer^35^ show the ejection of silk at the silk-ejector in a specimen fixated during the spinning process. Even though the individual structural elements of the spinning apparatus have been known for some time only the three-dimensional reconstruction unequivocally shows that a single gland with its reservoir and “canal cage” is connected via a single duct to a single silk-ejector (spinning bristle).

Beside the sexual differences mentioned by Edgerly et al.^28^, the spinning apparatus of the Embioptera species investigated shows distinct interspecific differences. The oligotomid species that live on trees have fewer than 100 reservoirs while those species living on the ground have distinctly more than 100 reservoirs. Even the opportunistic *O. nigra* and *E. auripecta* fit into this pattern. They have reservoir numbers about 100, apparently intermediate between the two groups. This pattern is also confirmed by Barth^32^ who found 180 glands (and therefore reservoirs) in both tarsi (consequently 90 reservoirs per tarsus) in the tree dwelling *Archembia batesi* (MacLachlan, 1877). The exception to this pattern is the clothodid *An. urichi* (serving as an outgroup), a tree dweller having 152 reservoirs. In the large-bodied tree dwelling Embioptera (*An. urichi* and *E. auripecta*) the reservoirs are more voluminous than in the ground dwelling species. The existence of more gland tissue in the ground dwelling species, the embiids with the smaller reservoirs, could lead back to the lower storage capacity. Hence, these embiids probably have to replace the silk constantly and at a higher rate to provide a constant spinning result.

Following Sehnal & Akai^39^, insect silk glands can be of three different types: labial, Malpighian tubule, or dermal glands. The embiopteran spinning apparatus most likely can be categorized as dermal glands with class III secretory units^9^. Diagnostic features of this homology are that the duct is lined with the basal lamina of the gland hypodermis, and degenerated nuclei, described as “Kanalzellen”^32^, can be found along the duct. At the distal end of the duct, the basal membrane lines the cuticular silk-ejectors nearly to its end. Entering the reservoir the duct forms a so-called “canal cage”^32^ (Fig. 4C), a complex structure that shows gross similarity with the end-apparatus of other class III dermal glands^9^. The homology of the glands found in Embioptera with class III dermal glands is therefore most likely.

In Arthropoda spinning apparatusses are well known. The most complex and arguably best investigated spinning apparatus is that of the spiders (Arachnida: Araneae) where the silk is produced by a complexity of glands and spigots^40^,^41^,^42^,^43^,^44^,^45^. However, silk producing organs also found in other groups of arthropods like Pseudoscorpions, Acari and myriapods^45^ – produced by salivary (labial) glands, collateral (genital) glands and Malpighian tubules^46^. Due to the different morphological organisation and the distant position of the groups in the phylogenetic system of Arthropoda, a homology of the silk glands can safely be excluded^47^,^48^,^49^.

Silk glands found in other insects, such as the Malpighian tubules that are often used for producing a cocoon for pupation^9^ and labial silk glands, which function in many groups in the larval stage and produce silk in connection with metamorphosis^8^,^9^,^50^, have no relationship, evolutionarily or morphologically, to the tarsal glands of Embioptera.

Dermal silk glands occur in bristletails and silverfish, water beetles, lacewings, parasitic wasps, non-parasitic wasps, dance flies as well as in webspinners^9^,^28^. Dermal silk glands, which comprise a variety of silk producing apparatuses in a variety of insect groups, all share similarities to sense organs. They can be isolated units or clustered together; they are associated with the external cuticle (epidermal) or with cuticular structures inside an insect body (hypodermal). These observations led some authors to propose that dermal silk glands and sense organs could be homologous^9^,^51^,^52^. The morphological variability and the usage of class III dermal glands are diverse. E.g., in sphecid wasps the females use silk produced by their dermal glands located on their sternum, for nest lining^53^,^54^. Also very common is the association of dermal silk glands with the reproductive system of females. In this context, the glands often produce additional secretion for nutrition, egg coating, glues and pheromones^9^. In viviparous flies dermal silk glands provide the nutrition for their larvae^55^. Silk production by dermal glands, however, is not always associated with the female genitalia: male Thysanura, for example, have a complex spinning apparatus^47^. Furthermore, mating behaviour can be related to the silk spinning process as in male hilarine or dance flies (Empidinae). The male of these flies tend to use their basal tarsomeres to secrete silk from ventrally located spinning bristles. This tarsal silk is used to bunch together algae and prey insects as a mating present for females^7^. The spinning apparatus is only present in the fore-tarsi of the males and it is composed of 12 pairs of glands each connected via a single duct to a spinning bristle^7^. The spinning apparatus of webspinners and dance flies may be of similar developmental origin as already mentioned by Collin et al.^37^, because of a very similar construction and location. However, the number of silk glands is distinctly different. The spinning apparatus of Embioptera can be composed of over hundred silk glands, whereas the hilarine flies show only ca. 12 pairs of silk glands^7^. Furthermore, the usage of the silk and therefore the composition is distinctly different; hilarine males use their silk to rope up algae from the surface of freshwater creeks. Those small parcels serve as pre-mating gifts to females^7^. In contrast, Embioptera produce thick and protective galleries in which they dwell. Due to the distant phylogenetic placement of hilarine flies and embiids within the insects, a direct homology of the similarly built silk glands in the basitarsomeres of both groups can be excluded. However, in both cases class III dermal silk glands probably have been used to build the spinning apparatuses. This assumption is supported by our indications of the presence of a characteristic end-apparatus (“canal cage” in Embioptera) in each individual gland.

The present comparative study of morphometrics, behaviour and body size uncovered patterns in a complex dataset by integrating morphological characterization of the silk glands, an analysis of spinning behaviour, and two distinct statistical approaches with independent contrast analysis. Statistical tests highlighted variables that related to variation in body size for these insects that rely to varying degrees on silk for protection. For example, significant differences appeared in gland structure, such as reservoir volume, and the length and width of the basitarsomere (the spinning tarsus) and these were correlated with body size. These correlations highlighted the potential productivity of the glands because more voluminous reservoirs were found in the larger webspinners that might rely more on copious silk to cover their bodies with silk. No correlation was detected between independent contrasts for the spinning behaviour measurements and body length. However, the reservoir volume differences could account for greater ability to produce silk with each spin step. Unquantified observations by JSE align with this proposal because when *An. urichi* and *E. auripecta* spin silk it is clearly visible to the eye, even after a short bout of spinning. In contrast, when *H. solieri* or the other small species spin, very little silk is visible even after hundreds of spin steps. When the length and width of the spinning tarsus is corrected for body length, this structure in the smaller webspinners appears relatively longer and wider than what is found in the larger insects. Perhaps there is a minimum size needed to effectively produce the silk, giving the smaller insects the order-defining trait of having disproportionally big front feet. Possibly if the spinning tarsus increased proportionally with body length, the other function of front legs (locomotion) might be compromised. Ongoing investigation of one of the larger webspiners, *An. urichi,* using high-speed videography, has shown that her spinning tarsi appear to slip when the insect is running quickly backwards, as they tend to do when escaping a threat (unpublished observations, JSE). For webspinners, a trade-off between efficient locomotion and investment in silk glands is likely and perhaps increasing the volume of the reservoirs allows for greater production of silk despite the relatively smaller tarsal size in the larger webspinners.

Collin and colleagues^37^ suggested a positive correlation between body length and the number of reservoirs. This hypothesis was based on the assumption that the number of ejectors per tarsus is equivalent to the number of reservoirs. In general, this assumption is correct; however, Collin et al.^37^ only used scanning electron microscopy for counting the setae on the surface of the tarsus. This lead to a miscalculation of the number of glands (230 instead of our 152 glands for *Antipaluria*, Tab. 1), because not every seta on the spinning tarsus is a silk ejector, which is difficult to recognize in SEM images. The data we collected through counting the reservoirs inside the tarsus do not support a correlation between body length and the number of glands or reservoirs.

**Figure 7:**
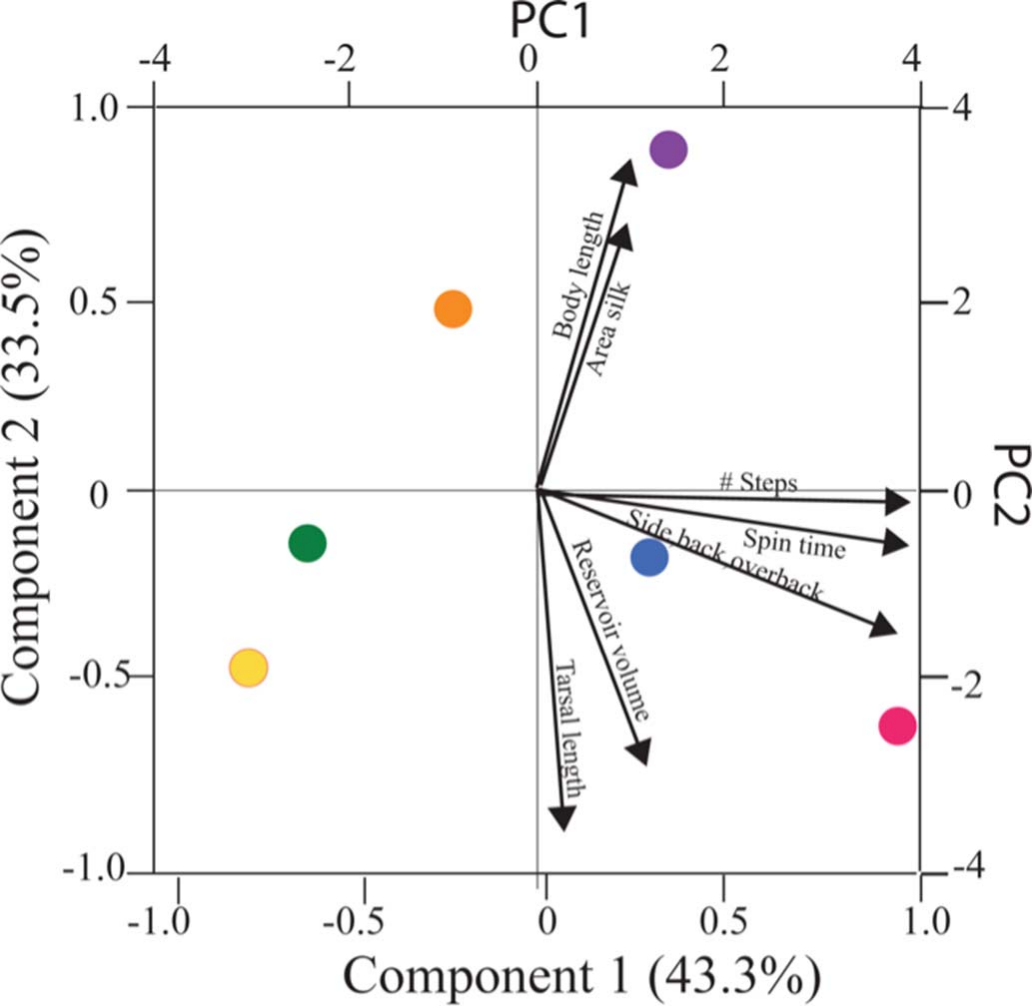
Principle component loadings based on analysis of independent contrast scores. Coloured points refer to nodes of the branching diagram in Fig. 3. Those vectors of magnitude 0.4 or greater are drawn. Table 2 shows eigenvector values for PC1, PC2 and PC3.

Principal components analysis revealed a pattern in the overall dataset that included behavioural details as well as structural. As predicted, the taxa with the smaller species (the green, orange and yellow nodes in Fig. 7) seem to invest less in silk: exhibiting fewer spin steps, less time spent spinning and fewer steps to the side, back and overback (which contribute more to tube construction). The magenta-coloured node representing the genus *Haploembia* did not fit this pattern. One of the species in this genus, *H. solieri,* tended to spin for long periods of time and with complex stepping. Its congener expressed the opposite, and in fact, was very reluctant to spin at all. Clearly more needs to be learned to determine how silk spinning diversity has evolved in this order. Our analysis of seven species has hinted that larger species may be more likely to invest in complex spinning than smaller ones. However, a broader study is required to determine the generality of these findings. How the fine structural features of the glands, visible in Supplemental Fig. 2 and 3, specifically relate to silk productivity also is still not understood.

Previous work on webspinner silk has shown that the fibroin proteins themselves are very similar and highly conserved^37^. Four of the genera (*Antipaluria, Oligotoma, Aposthonia, Haploembia*) in our study were included in the survey of webspinner silk conducted by Collin et al.^37^. Our current work demonstrates that the reservoirs of the silk-producing glands vary in interesting ways. But again, how these variations relate to silk production remains a question yet to be solved.

The seven species selected for this analysis are closely related except for the one selected as an outgroup, *An. urichi.* The selection was strategic because we wished to test webspinners that had a close evolutionary relationship but varied in attributes that might be related to their reliance on silk or silk spinning behaviours. While the sample size is small, we were able to detect significant relationships and differences between species. Expanding the sample clearly would help clarify the relationships even more. We note that having six related species of Embioptera to investigate was hard-won, because of the challenges associated with locating them in the field, rearing them in the laboratory, and filming them so that we could score their behaviours.

The clearest pattern discovered for the seven species in the set is that those that rely more on silk when creating their domiciles appear to invest in more voluminous silk reservoirs, even when corrected for body length. The behavioural repertoire associated with spinning did not appear to vary much and did not relate to details of gland morphology.

## Methods

We evaluated silk spinning behaviour and silk gland morphology of *Antipaluria urichi* Saussure, 1896 (Clothodidae), as an outgroup, in comparison with six species of Oligotomidae: *Aposthonia borneensis* Hagen, 1885, *Aposthonia ceylonica* Enderlein, 1912, *Eosembia auripecta* Ross, 2007, *Haploembia solieri* Rambur, 1842, *Haploembia tarsalis* (Ross, 1940) and *Oligotoma nigra* Hagen, 1866 (Supplementary Fig. 1). These species cover a range of body sizes and lifestyles and should vary in how conspicuous adults are to potential natural enemies and how exposed to the elements they are. The number of species investigated is relatively small, partly because of the difficulty in finding and rearing these insects, and because of the high expense of imaging their silk glands. All specimens used in this study originate from breeding cultures housed in the Department of Biology of Santa Clara University, USA. All regulations concerning the protection of free-living species were followed.

### Gland Morphology

Two adult females of each species were fixed in an FAE solution^56^ and subsequently stored in 70% ethanol. Prior to µCT investigation specimens were dried with a Balzer CPD 030 critical point dryer. Gland morphology was investigated with synchrotron radiation micro computed tomography (SRμCT). The data were generated at the Deutsches Elektronen Synchrotron (DESY) in Hamburg (Germany), beamline Petra III, Proposal no. I-20090102, Aug. 2009, SB, at the Swiss Light Source (SLS) in Villingen (Switzerland), beamline Tomcat, Proposal no. 20080794, Mai 2009, ThH and at the Helmholtzzentrum Berlin (BESSY II) in Berlin (Germany), beamline BW2, Proposal no. 2009_90372, Aug. 2009, SB. Three-dimensional reconstruction, processing and visualization of the data were done with Amira® 5.4 (Visage Imaging). Measurements were also done with Amira®, which allows a highly accurate assessment of volumes and lengths.^57^,^58^,^59^ Images of the reconstructions were subsequently processed in Photoshop CS3 (Adobe System Inc.). The output unit of Amira® for measurements (number of volume-pixel = voxel) was converted into cubic millimetre (mm^3^) and millimetre (mm). For reasons of comparison length and volume data are also provided in micrometer (μm) and cubic micrometer (μm^3^) (Table 1). A voxel is a volume pixel with discrete values of XYZ-coordinates within the dataset. The spatial resolution was determined by the beamline parameters and the lens applied during data acquisition.

### Silk Spinning Behaviour

Three aspects of silk spinning behaviour were quantified in the laboratory at Santa Clara University: silk production, how much time is devoted to silk spinning, and details of silk spinning behaviour. Silk production was evaluated by placing groups of four adult females into a circular-shaped plexiglass arena (20 mm diameter × 4 cm tall) with a flat, black, textured base where they could spin silk. Because silk is white, the black background provided contrast for photographs taken after 24 hours of spinning in the arena. Photographs were taken with a Canon Eos Digital Rebel camera and the area of silk was analyzed with ImageJ Freeware (see^28^ for more details). The area of silk was compared in terms of total weight (g) of the females in the group. Number of replicates varied from one to four (see Table 1 for sample sizes).

How much time was devoted to spinning and the details of silk spinning were determined by placing solitary adult females in arenas, filming them for one hour, and establishing a time budget for each. Behaviour acts, recorded with Observer software (version 5.0, Noldus Information Tehnology,), ranged from spinning to traveling to grooming, as well as others not relevant to the current focus. When a webspinner spins silk it displays characteristic steps around its head and body. These steps were named for their relative positions such as near the head, reaching to the side of the head, along the side of the thorax and near the abdomen and over the dorsum of the abdomen (called back and overback respectively). Other steps included crossing the sternum from one side to the other and other variations of stepping around the front end of the body. See Edgerly et al.^28^ for detailed illustrations of silk spinning steps that reflect the procedure applied herein for quantifying step diversity. Individual webspinners were placed in either a burrow apparatus, consisting of a groove (0.3 cm wide × 0.5 cm deep × 5.8 cm long; Supplementary Fig. 4A; Supplementary Fig. 4 shows sample sizes) drilled into a plywood block, or into a plexiglass chamber (inner dimension of 6 × 6 × 6 cm) lined on one side with oak bark (Supplementary Fig. 4C). The two arenas were suspended in front of a video camera for filming (Supplementary Fig. 4B). DVDs were analyzed in slow motion playback while one observer called out the spin steps as another entered the information into the Observer event recorder. Spinning details were summarized as mean number of steps, proportion of steps to the side, back and overback, and overall diversity of steps (computed by applying a modification of the Simpson’s Diversity Index (inverse of dominance) as an expression of diversity^60^). The prediction was that greater diversity of spinning would reflect a greater reliance on silk because inclusion of more steps over the dorsum are necessary for creating body-enclosing silk tubes.

Time spent spinning in the burrow and chamber–style arenas were compared to determine if webspinners express spinning more or less in situations that resemble their native microhabitats. As such, arboreal species might spin in the open chamber whereas crevice dwellers might not, and vice versa when exposed to the burrow arena. As expected, particular species were not inclined to spin fully or even at all in one of the arenas (Supplementary Fig. 5). To reflect as much as possible a realistic expression of a species’ silk spinning style, the details of spin steps for a species were based on the behaviour elicited in whichever arena triggered more complex spinning—for *An. urichi* and *Ap. borneensis* that was the chamber–style arena; for all others it was the burrow (see Table 1 for sample sizes).

### Test of Independent Contrasts

A test of independent contrasts was used to assess whether characteristics of spinning behaviour and the differences in morphological investment in silk production are related to body length. This test corrects for the phylogenetic relationships among species. The test requires accurate topology of a phylogeny containing the species of interest as well as the assumption that the character traits of interest express Brownian motion^61^. We adapted a phylogeny of the focal species from the known phylogeny of Embioptera. This phylogeny was developed using molecular data from 5 genes (16S rRNA, 18S rRNA, 28S rRNA, cytochrome oxidase I and histone III; 6844 bp) and 95 morphological traits^27^. The adapted phylogeny (Fig. 3) was used to perform a test of independent contrasts in the program Mesquite^62^, branch lengths on the tree were set to 1. Table 1 shows the behavioural and morphological data. We performed linear regressions on six morphological and five behavioural traits (using (PDAP^63^)) to test which were dependent or independent of the species’ average body length. We used a Bonferroni correction to control overall Type 1 error rate associated with performing multiple comparisons. In addition, to further explore how silk gland morphology and behaviour might co-vary, we conducted a principle component analysis (PCA) on the independent contrast scores of the three morphological measurements found to be significant and the spinning behaviours using JMP Pro 10 Statistics Program by SAS Institute Inc.

## Author Contributions

S.B., T.H. and J.S.E. designed research and developed the concept of the paper. S.B. carried out the morphological studies. J.S.E. performed the behavioural experiments. K.H. and D.M. performed the independent contrast analysis. S.B., T.H., D.M. and J.S.E. wrote the manuscript. All authors read and approved the final manuscript.

## Supplementary Material

Supplementary Information Supplementary Figures

## Figures and Tables

**Table 1:** Morphological and behavioural measurements related to silk glands and silk spinning in Embioptera*.

Species (Body length) (mm)	Resolution	Log Volume (mm^3^) Tarsus	Log Length (μm) Tarsus	Log Width (μm) Tarsus	Log Ø Volume (μm^3^) Reservoirs	# Reservoirs	Log Volume (μm) Silk Gland	Log Area Silk Spun in 24 h (Mean) (# Replicates)	Mean Spin Step Diversity Index	Log Mean # Spin Steps in 1 h trial (# Replicates)	Log Mean Time Spent Spinning (s)	Mean % Side, Back & Overback of Total Spin Steps
*Haploembia tarsalis* (9.5)	0.370 μm	1.5913	2.1648	2.1022	4.0748	144	7.383	4.4623 (2)	3.431	3.0402 (3)	1.5854	0.437
*Haploembia solieri* (9.5)	0.370 μm	1.2847	2.1245	1.9106	3.8502	174	7.558	4.2041 (1)	4.327	3.7611 (6)	3.1051	16.163
*Aposthonia ceylonica* (7.5)	0.370 μm	1.3481	2.1570	1.9374	4.2334	53	7.235	6.009 (4)	6.552	3.2819 (4)	2.7291	14.004
*Oligotoma nigra* (11.0)	3.614 μm	1.6081	2.1843	1.9140	4.5452	111	7.285	4.944 (2)	4.350	3.3953 (4)	2.8733	1.470
*Aposthonia borneensis* (11.5)	0.370 μm	1.4188	2.2557	2.00595	4.8167	83	7.438	5.0334 (4)	5.682	3.3546(5)	3.0441	11.812
*Eosembia auripecta* (20.0)	3.614 μm	1.8729	2.3214	2.0144	6.0169	91	7.812	5.7626 (1)	3.529	2.7732 (4)	2.3222	13.790
*Antipaluria urichi* (22.0)	3.614 μm	1.7837	2.3169	2.0493	5.9734	152	7.379	5.2068 (4)	6.472	3.4448 (5)	3.1411	20.480

*two specimens were imaged and the mean is shown for each morphological measurement

**Table 2:** Eigenvectors from PCA of independent contrasts of silk gland characters and spinning behaviours for seven species of Embioptera. Two principal components account for 77% of the variation, three for 93%. See Table 1 for details of morphology and behaviour measurements. PC1 and PC2 are significant at *P* < 0.001 and 0.018 respectively.

Characters	PC1 (43.26%)	PC2 (33.46%)	PC3 (16.36%)
Body length	0.11186	**0.50530**	−0.31087
%Side, back, overback	**0.45690**	−0.20846	0.05665
Spin step diversity	0.36835	0.02181	−**0.55665**
Log (Mean # spin steps)	**0.49541**	−0.01374	−0.09609
Log (Time spent spinning)	**0.49496**	−0.08436	0.11608
Log (Area silk spun)	0.09809	−**0.41059**	0.34430
Log Tarsus length/body length	0.03255	−**0.50382**	−0.30665
Log Tarsus width/body length	0.35077	0.31850	0.31596
Log Reservoir volume/body length	0.13751	−**0.41059**	**0.50514**
