# Supplementary material for: The spinning apparatus of webspinners – functional-morphology, morphometrics and spinning behaviour
Source: Sci Rep. 2015 May 7;4:9986. doi: 10.1038/srep09986 (PMC4423565; doi:10.1038/srep09986)
Supplement: Supplementary Information — Supplementary Figures [file srep09986-s1.pdf]

Supplementary Figures

for „The spinning apparatus of webspinners – functional-morphology, morphometrics and spinning behaviour"

by Sebastian Büsse, Thomas Hörnschemeyer, Kyle Hohu, David McMillan & Janice S. Edgerly

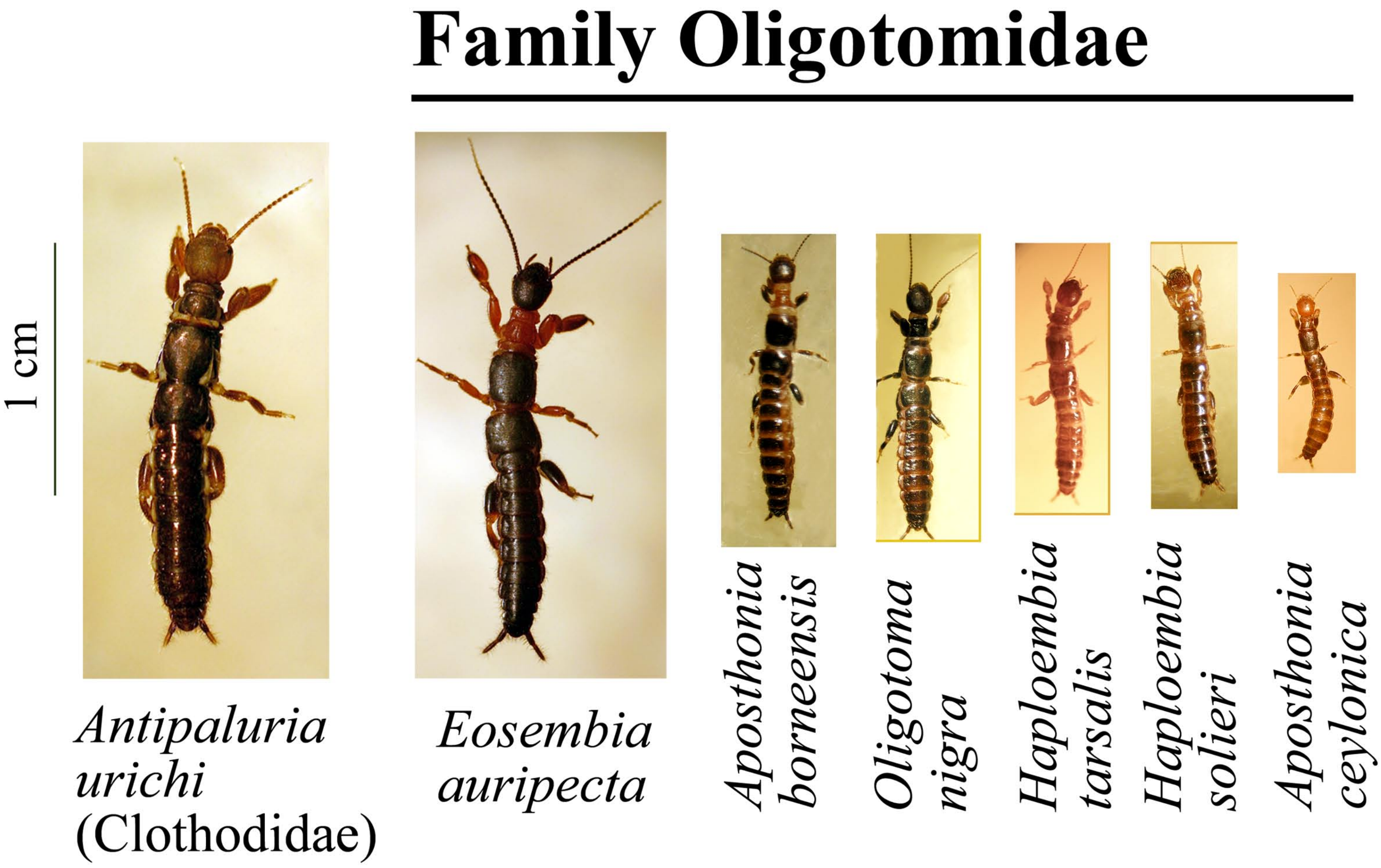

Supplementary Figure 1. Adult females of the focal species evaluated for silk glands and silk spinning behaviours. All are in the family Oligotomidae except for *An. urichi* of the famliy Clothodidae, selected as an outgroup for this work. The scale line shows the approximate size of these individuals. All photographs were taken of living specimens in the lab at Santa Clara University.

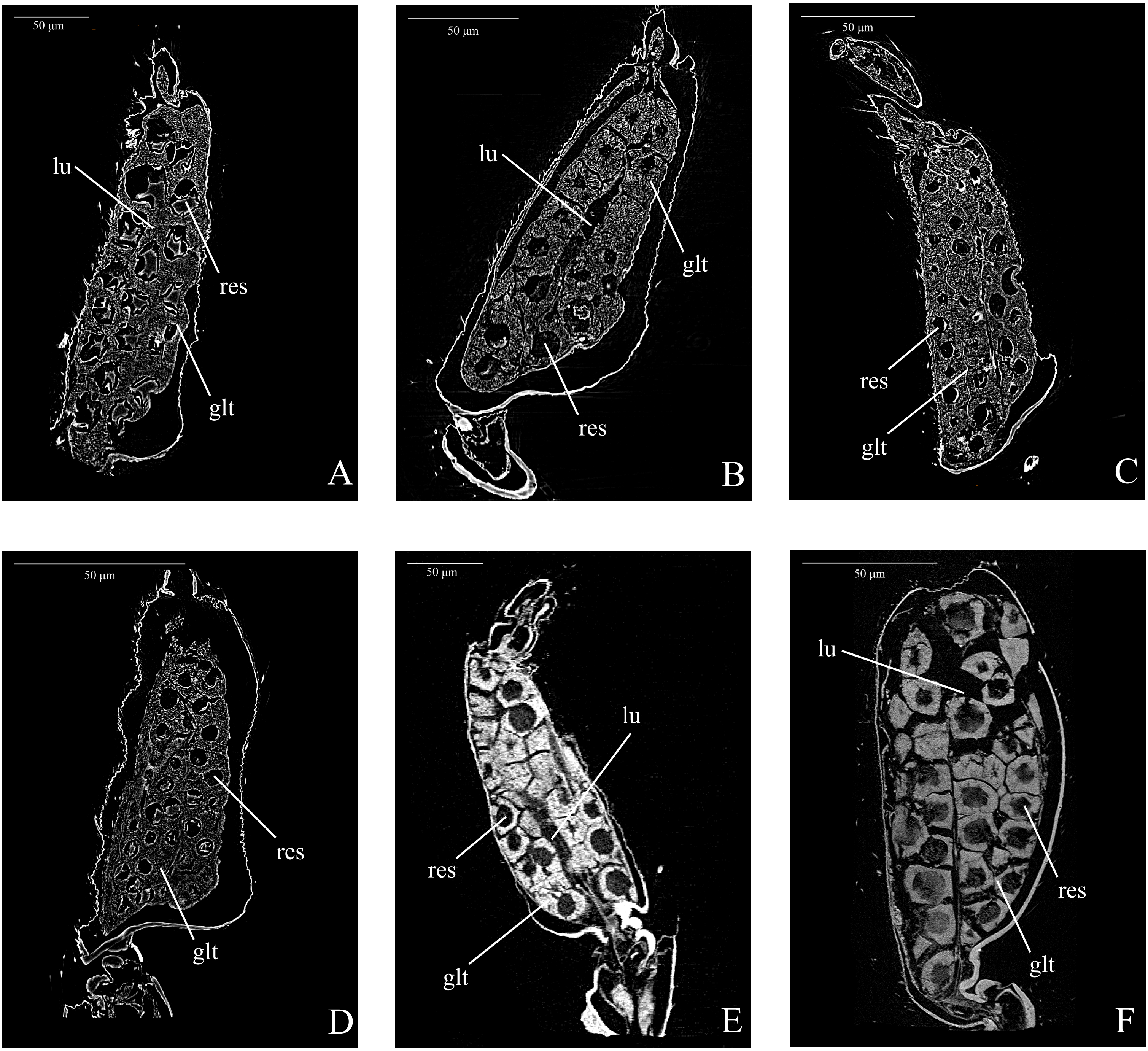

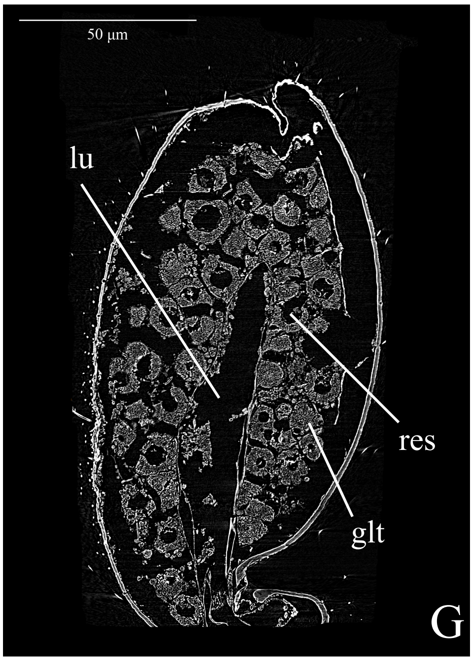

Supplementary Figure 2: A-G Longitudinal sections through prothoracic basitarsomer using SRµCT. Glt - gland tissue; lu - lumen; res - gland reservoir

A. *Aposthonia borneensis*. B. *Aposthonia ceylonica*. C. *Haploembia solieri*. D. *Haploembia tarsalis*. E. *Oligotoma nigra*. F. *Eosembia auripecta*. G. *Antipaluria urichi*.

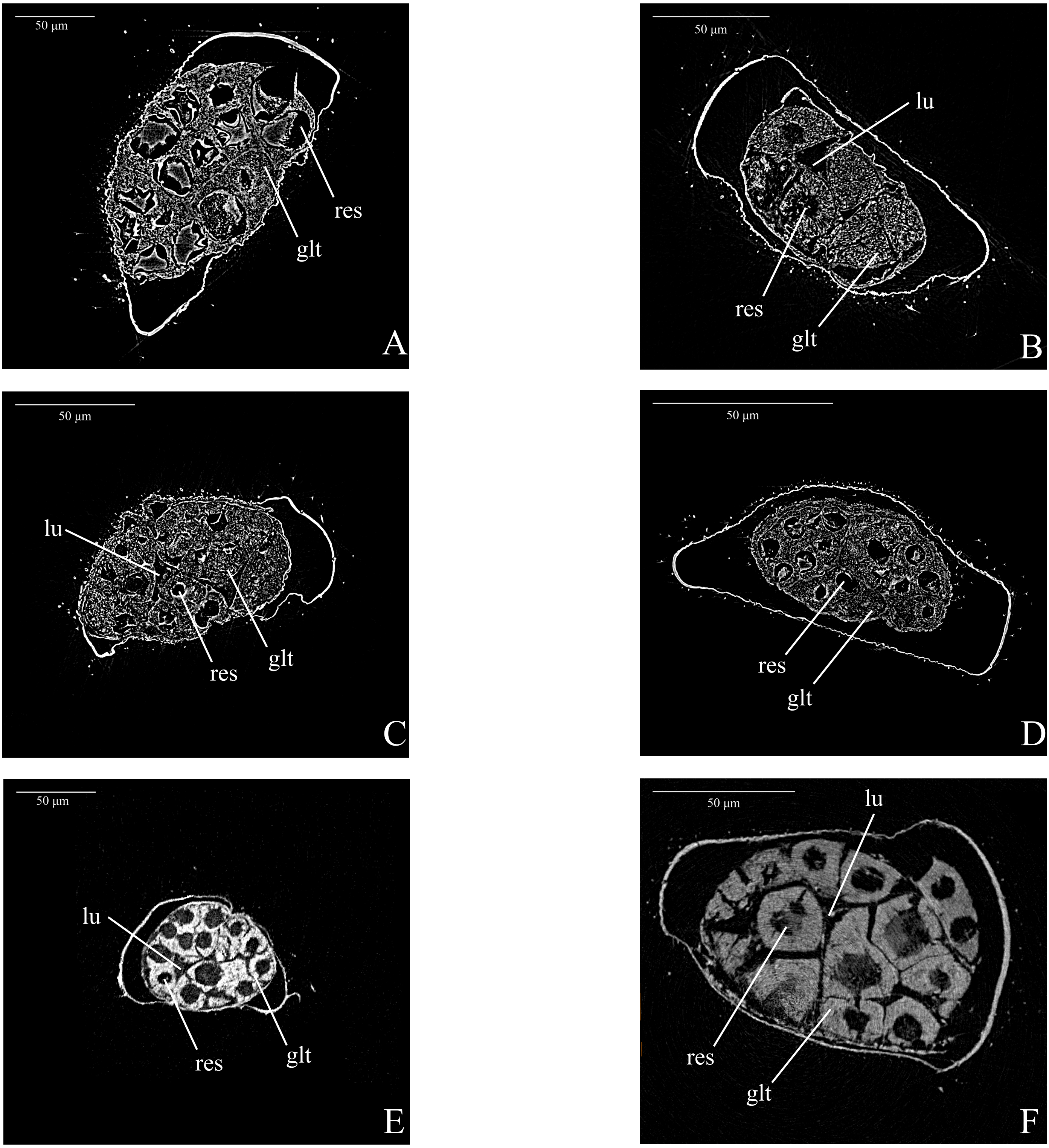

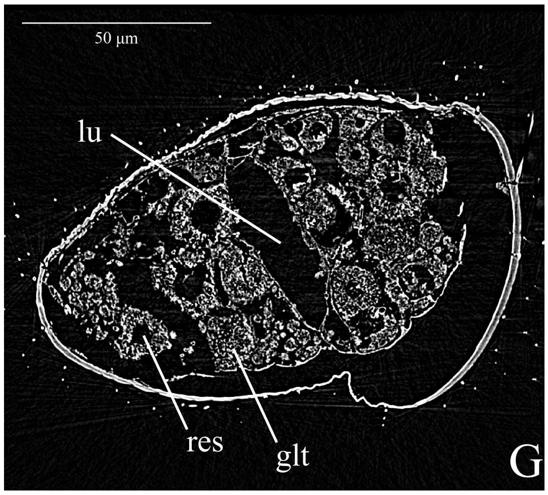

Supplementary Figure 3: A-G Cross sections through xprothoracic basitarsomer using SRµCT. Glt - gland tissue; lu - lumen; res - gland reservoir

A. *Aposthonia borneensis*. B. *Aposthonia ceylonica*. C. *Haploembia solieri*. D. *Haploembia tarsalis*. E. *Oligotoma nigra*. F. *Eosembia auripecta*. G. *Antipaluria urichi*.

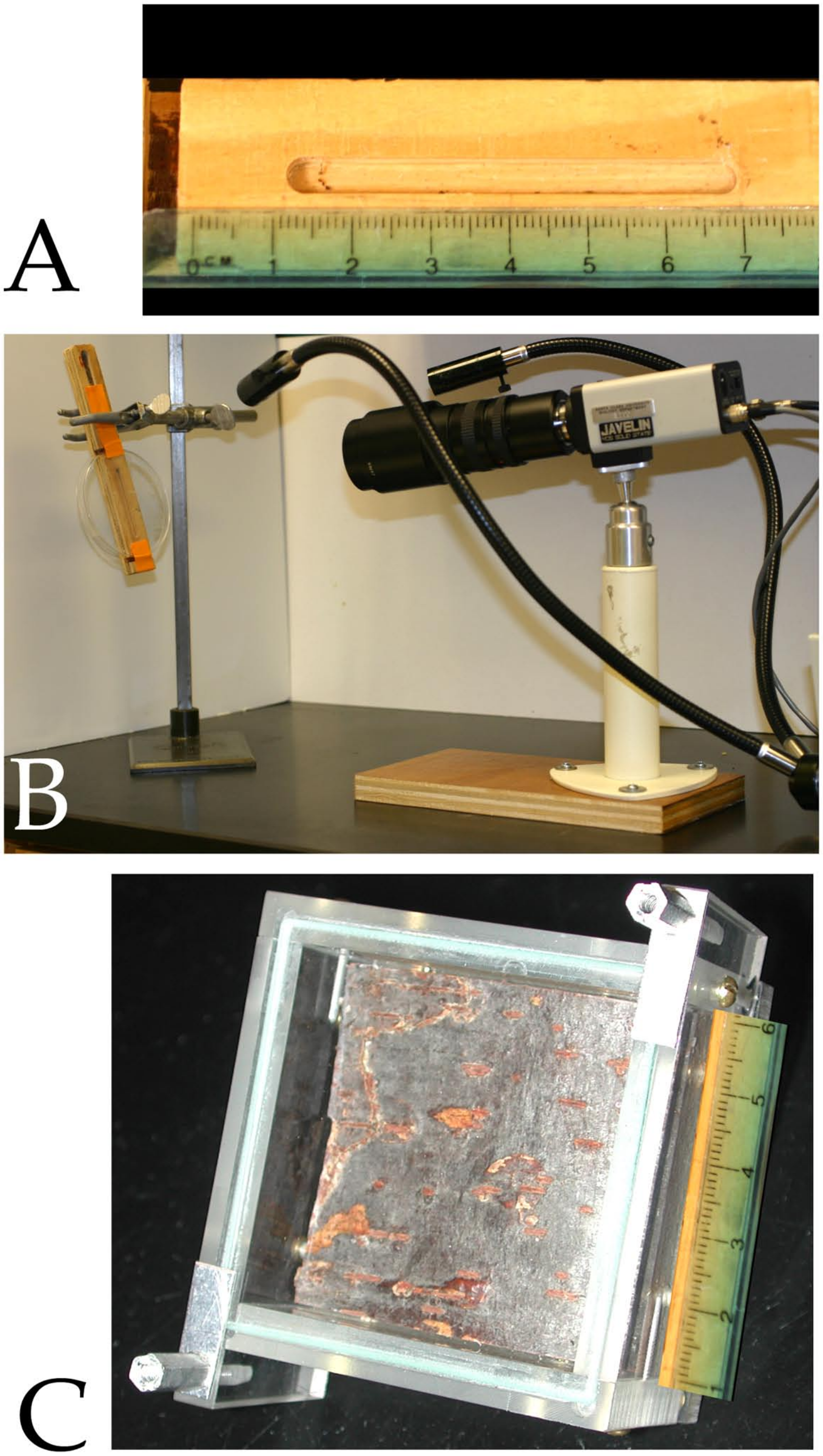

Supplementary Figure 4. Arenas used to stage silk spininng bouts, filmed and scored.
(A) A burrow (0.3 cm wide X 0.5 cm deep X 5.8 cm long) carved into a wooden block.
(B) The video camera and lighting set-up. (C) The chamber arena (6 X 6 X 6 cm) lined with bark on one side.

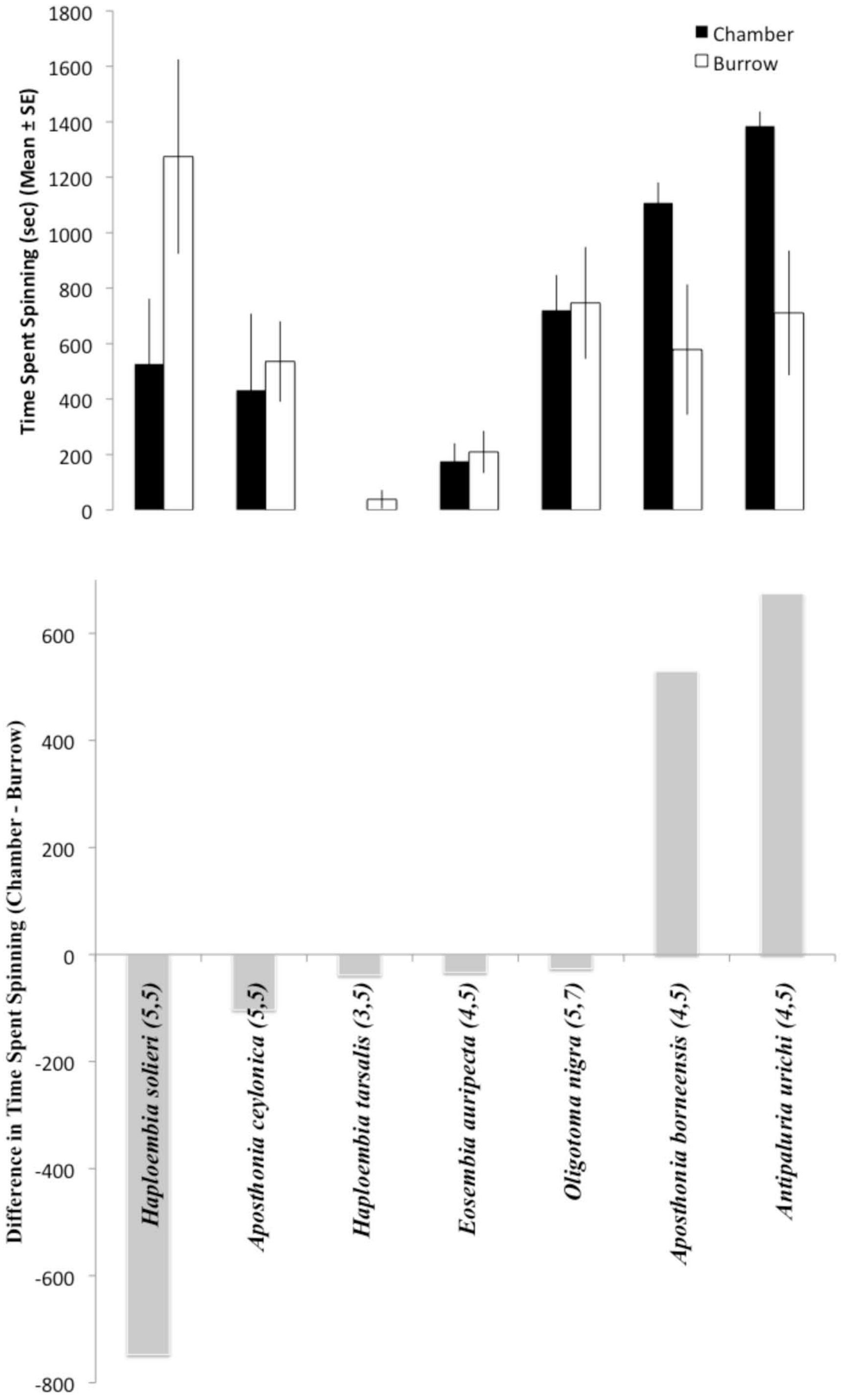

Supplementary Figure 5. Time spent spinning in the different arenas, burrow or chamber, for the seven focal species. Individuals of these species showed differential responses to the arenas; some would spin more in one than in the other type. For example, the ground-dwelling webspinner *H. solieri* spun in both arenas, but spent much more time spinning in the burrow. *Antipaluria urichi* spent more time spinning in the chamber, which resembles their natural habitat of tree bark. (Top) Mean times spent spinning in the chamber and the burrow. (Bottom) Differences in spinning between the two arenas. Number of individuals tested are shown in parentheses next to each species' name as "# in the burrow, # in the chamber". These results were used to determine which arena triggered the most spinning by individuals of a species. The spinning style for a species was quantified based on videos of females in the more-preferred arenas as we attempted to obtain a more complete picture of the choreography of spinning. Even if the preference was not obvoius (as for *O. nigra* and *E. auripecta*), we used information based on the arena where more spinning occurred. This is important because if an arena did not trigger much spinning, the individuals were not expressing all the steps that they might in their natural environment. See Table 1 for sample sizes used for further analysis in this study.
